# Dataset on the tested and simulated response of thick cold-formed circular hollow sections under cyclic loading

**DOI:** 10.1016/j.dib.2024.110122

**Published:** 2024-02-01

**Authors:** Adam Jan Sadowski, Wei Jun Wong, Sai Chung Simon Li, Christian Málaga-Chuquitaype

**Affiliations:** a Department of Civil and Environmental Engineering, Imperial College London, UK; b Faculty of Mechanical Engineering, Delft University of Technology, the Netherlands

**Keywords:** Cyclic loading, Seismic design, Circular hollow section, Digital image correlation, Kinematic hardening, Finite element analysis

## Abstract

This article describes a dataset used to calibrate a finite element model of a thick circular hollow section (CHS) with varying *d*/*t* (diameter to thickness) ratio under cyclic loading which may be used as a computational model validation benchmark by researchers working on similar problems in structural and mechanical engineering. The test data consists of seven cold-formed S335J2H steel CHS tube specimens tested to buckling failure in low-cycle fatigue under a three-point bending arrangement, instrumented with discrete strain gauges, displacement transducers and string potentiometers together with continuous surface deformation fields obtained by two pairs of digital image correlation (DIC) cameras. ‘Half-cycle’ material data from the uniaxial tensile testing of dog-bone coupons is also provided. Comparisons between measured and simulated entities such as midspan forces, moments, displacements and mean curvatures can be obtained with MATLAB processing scripts. Complete ABAQUS model input files are also provided to aid in benchmarking.

Specifications Table

**Table:** 

Subject	Civil and Structural Engineering
Specific subject area	Seismic design of tubular steel structures, design rule development
Data format	Analysed (material tensile tests processed to correct for the double curvature of the dog-bone coupons, DIC measurements processed to obtain neutral axis curvatures, post-processed finite element simulation data)
Type of data	.xlsx file (Table, Chart) – dataset with analysis and figures to aid in interpretation .st and .dic files (Table) – ASCII text files containing formatted numerical data (the choice of file extension is merely for convenience) .inp files (Other) – ABAQUS finite element software input files .png and .fig files (Figure) – Stand-alone Figure files illustrating the data .m file (Code) – MATLAB script for automated processing and visualisation
Data collection	Uniaxial material tensile testing was performed following standardised procedures (e.g. ASTM A370-14) with an Instron 250 kN 8802 dynamic machine, supplemented by uniaxial strain gauges and geometry measurements by digital Vernier callipers. The raw data was processed to correct for the load eccentricity caused by the double-curvature of the dog-bone specimens. Cyclic three-point bending tests were performed in a custom-built rig with a displacement-controlled loading protocol introduced by an Instron hydraulic actuator, together with uniaxial strain gauges, string potentiometers and a two-pair (four-camera) digital image correction (DIC) system. The DIC data gathered continuous surface deformation fields in the vicinity of the midspan plastic hinge, subsequently processed with the DaVis v8.4 software and custom-written MATLAB code to approximate the local curvature of the neutral axis at this location. Finite element simulations were performed using the ABAQUS commercial software to reproduce the cyclic three-point bending tests which assumed perfect cylindrical geometries but used a kinematic strain hardening model calibrated as far as was possible on the uniaxial ‘half-cycle’ material tensile test data. Test vs. simulation comparisons appear acceptable and may be used for model validation and benchmarking purposes. MATLAB code was developed to facilitate this comparison.
Data source location	Structures Laboratory, South Kensington, Imperial College London, UK.
Data accessibility	Repository name: Figshare Data identification number: 10.6084/m9.figshare.24560773 Direct URL to data: https://figshare.com/articles/dataset/Dataset_on_the_tested_and_simulated_response_of_thick_cold-formed_circular_hollow_sections_under_cyclic_loading/24560773
Related research article	Sadowski A.J., Wong W.J., Li S.C.S., Málaga-Chuquitaype C. & Pachakis D. (2020) “Critical buckling strains in thick cold-formed circular-hollow sections under cyclic loading” ASCE Journal of Structural Engineering, 146(9), 04020179. 10.1061/(ASCE)ST.1943-541X.0002747

## 1 Value of the Data

The original research article [1] generated a significant amount of data [2] which is hereby being released in abridged form. Its value to the community is as follows:

• The stress-strain curves for cold-formed S355J2H tubes can be used by researchers as direct inputs into material models or combined with wider databases of stress-strain curves for the purposes of statistical characterisation [3].
• The cyclic bending test results may be used to supplement datasets of critical buckling strains of compact and semi-compact steel circular hollow sections (CHS) for the purposes of design rule development, following the spirit of Harn et al. [4]. Most bending tests of large-scale steel tubulars have historically been monotonic rather than cyclic, so this data contributes to filling an important research gap.
• The cyclic tests made novel use of digital image correlation (DIC) as a method of continuous surface deformation measurement to estimate midspan mean neutral axis curvatures and associated longitudinal critical buckling strains.
• The ABAQUS input files may be used as a validation benchmark (when compared against the tests data) and as a basis for researchers to build more advanced structural and material models of steel structural systems under cyclic loading.
• Where the dataset is used to validate computational models of individual CHS or thick tubulars, these can subsequently form the basis of models of more complex structures such as retaining quay walls or marine support structures such as piles subject to more complex loading conditions such as seismic acceleration records.
• Equally, the dataset may be used for the validation of simplified analytical models of idealised plastic hinges, appropriate for systems under non-uniform bending moment distributions.
• The communities likely to be most interested in this data and the supporting research [1] include civil, structural, geotechnical, mechanical and marine engineers specialising in earthquake engineering design of maritime structures, and in particular those practitioners involved in design rule development.

## 2 Background

The empirical and numerical datasets released here were compiled as part of a 2019 collaboration between Imperial College London and COWI Ltd, funded by a Research Award of the Institution of Structural Engineers (IStructE), exploring the inelastic behaviour of steel circular hollow sections under cyclic loading. The context was the evolution of the ASCE 61-14 American Standard on the seismic design of piers and wharves [5] which was taking place at the time under the auspices of the ASCE 61-19 Standard Committee, and the associated need to expand the empirical knowledge base on the ductile inelastic behaviour of steel hollow sections used as marine piles under low-cycle fatigue and likely critical strain limits for local buckling. Hitherto the majority of the tests and simulations had been performed under monotonic conditions, and it was intended that these new cyclic tests would inform parametric simulations to extend the range of coverage of updated design rules, and in particular provide more information on the dependency of the diameter-to-thickness ratio *D*/*t* on the critical bending strain. The original article [1] in which the dataset was first interpreted made a notable but limited contribution to this knowledge base owing to funding and timing constraints, and the dataset's release through this data article should give researchers the validation confidence to take this line of work further.

## 3 Data Description

The dataset [2] is distributed across a number of spreadsheets, folders and MATLAB processing scripts.

**Fig. 1:**
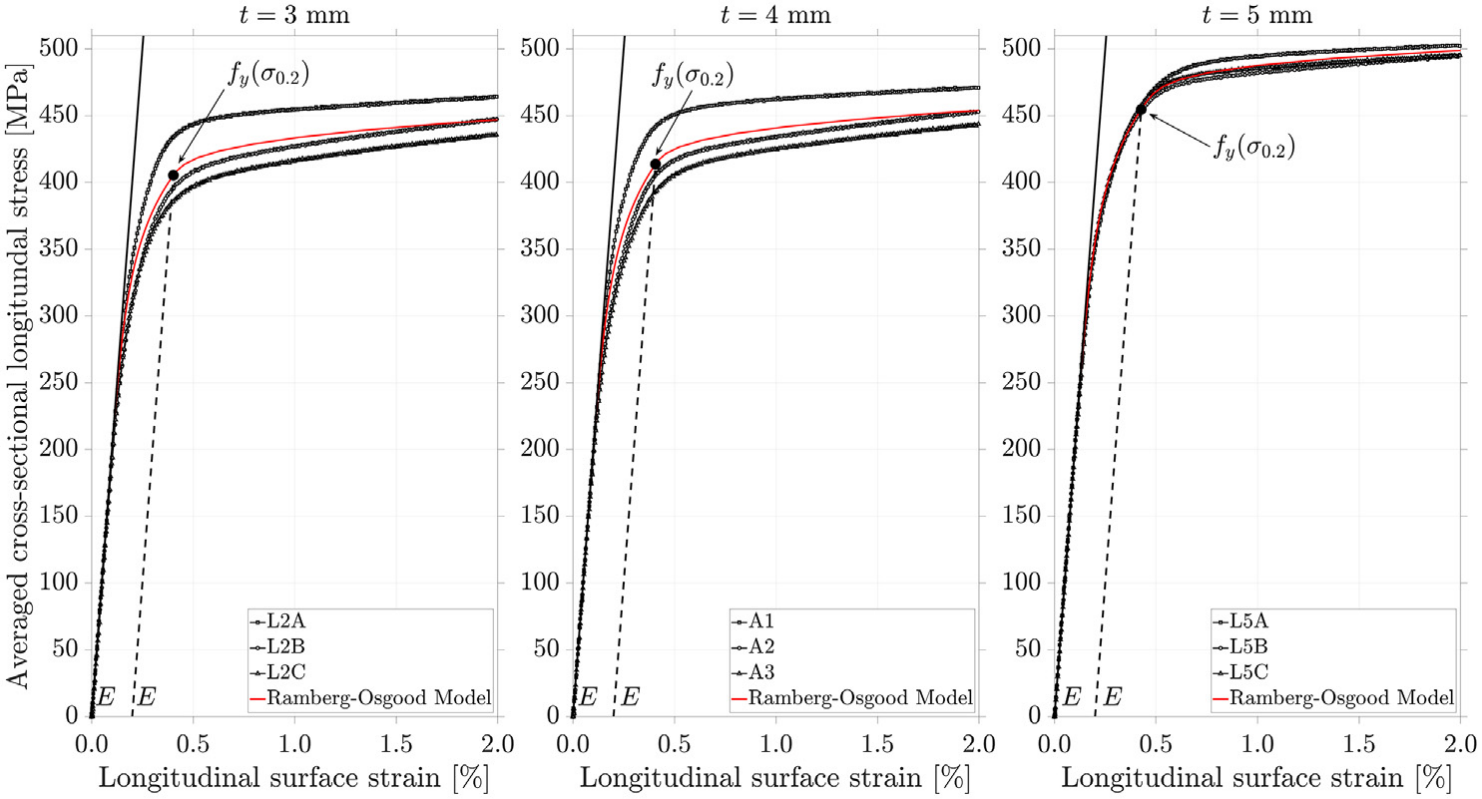
– Measured and fitted stress-strain curves for S355J2H cold-formed steel tubes of varying thickness. The figure includes an annotation of the initial tangent modulus *E* = 200 GPa and the yield stress taken as the 0.2 % proof stress *f_y_*(*σ*_0.2_) for the representative curve per nominal thickness.

The spreadsheet “Coupon Tests and Sectional Geometry.xlsx” in the root directory contains the complete data for tensile stress-strain curves of nine dog-bone specimens cut from cold-formed S355J2H tubes with nominal diameter-to-thickness *D*/*t* ratios between 28 and 47 (the choice of specimen dimensions being to maximise this *D*/*t* range as far as was possible within the constraints of the testing rig), three each from tubes with nominal thicknesses of *t* = 3, 4 and 5 mm. There is a general ‘Summary’ tab which additionally includes dog-bone specimen (and overall tube specimen) dimension measurements (as obtained by digital Vernier callipers), while each individual tensile test including processing required for a two-part Ramberg-Osgood fit [3] is contained within tabs ‘L2A’, ‘L2B’ and ‘L2C’ for *t* = 3 mm, then ‘A1’, ‘A2’ and ‘A3’ for *t* = 4 mm, and ‘L5A’, ‘L5B’ and ‘L5C’ for *t* = 5 mm (Fig. 1). The ‘FE model input’ spreadsheet presents the characterised stress-strain curves for *t* = 3, 4 and 5 mm based on averaged Ramberg-Osgood fit parameters and an initial tangent modulus *E* = 200 GPa. As these are in an engineering stress-strain format, they are transformed into the true stress-strain format (and specifically true stress – true *plastic* strain format) for consumption by the finite element solver. The remaining tabs are helpful figures. There was no particular justification for the dog-bone specimen naming scheme.

The repository contains three ABAQUS .inp files inside the “Abaqus input files” folder which can be submitted directly for analysis by the ABAQUS/Standard finite element solver. The file names are of the format “DYN_shell_t_X_L_2387_S4R.inp”, where X takes the value 3, 4 of 5 and represents the nominal thickness of the modelled tube specimen. Each ‘.inp’ file is a regular ASCII text file from which the geometry, material definition and displacement-controlled cyclic loading pattern (which followed the established protocol of Fulmer et al. [6]) may be easily extracted. Enough information should be available here to allow an analyst to reproduce the simulation using different software.

The folder “Processed test and simulation data” contains the three sub-folders and a number of MATLAB .m scripts. The “time_history_data” sub-folder includes numerous ‘.st’ and ‘.dic’ ASCII text files, designed so as to standardise the way the results of the cyclic bending tests and their simulations are presented for convenient direct comparison (they are inputs to a MATLAB script). As described shortly, ‘.st’ files contain time-history uniaxial strain gauge data (columns STT and STB correspond to L1 and L2 strain gauge positions respectively if to the ‘left’ of midspan, or R1 and R2 strain gauge positions if to the ‘right’ of midspan – Fig 0.2) while ‘.dic’ files contain, amongst other information, the time-history approximate position of the midspan tube neutral axis obtained from continuous optical capture with digital image correlation (DIC). MATLAB scripts present in this sub-folder include “process_st_dic_files.m” (and its associated “test_sim_object.m” class definition) to automate the processing of the ‘.st’ and ‘.dic’ files into informative time-history (e.g. midspan moment vs. time) and hysteresis (e.g. midspan force vs. midspan displacement) plots (if run, this will create the “individual_images” sub-folder which stores these plots), and “comparison_figures.m” to enable the direct comparison of tested and simulated relationships (and to create and populate the “comparison_images” sub-folder).

Lastly, for completeness and as a service to the community, the repository contains the entirety of the publicly available test data on critical buckling strains that served as an input to Figure 11 in the original paper [1], as inspired by a similar figure in Harn *et al*. [4], within the “Critical Strain Public Database.xlsx” spreadsheet. This is secondary data gathered by the Authors from the public domain, however its release complements the vision of this data article, it is fully referenced, refers to laboratory tests that were originally done on the basis of scientific principles and will be of value to researchers and design code developers working in this field.

## 4 Experimental Design, Materials and Methods

Readers are advised that the original article [1] already included significant detail on the experimental design and methods, and it is not intended to reproduce this information here. The discussion in what follows focuses on the computational validation aspect of the research.

The repository [2] describes three entirely distinct sets of data-gathering activities, both empirical and computational. The first activity was the programme of classical stress-strain tensile material testing of standardised dog-bone coupons machined from each one of the original tube specimens (i.e. three specimens each from tubes with *t* = 3, 4 and 5 mm). The deformation data was obtained from strain gauges placed at the midpoint of either side of the specimen and the tensile testing was performed using an Instron 250 kN 8802 dynamic machine. A full description may be found in the original paper [1], together with equations to correct for the effect of double curvature of the tube geometry and release of locked-in residual stresses after machining (both of which introduced a force eccentricity and thus a moment into the specimen). The data in the “FE model input” tab of the “Coupon Tests and Sectional Geometry.xlsx” spreadsheet already include this correction, but implementation details may be found in the individual tables labeled ‘L2A’ etc.

**Fig. 2:**
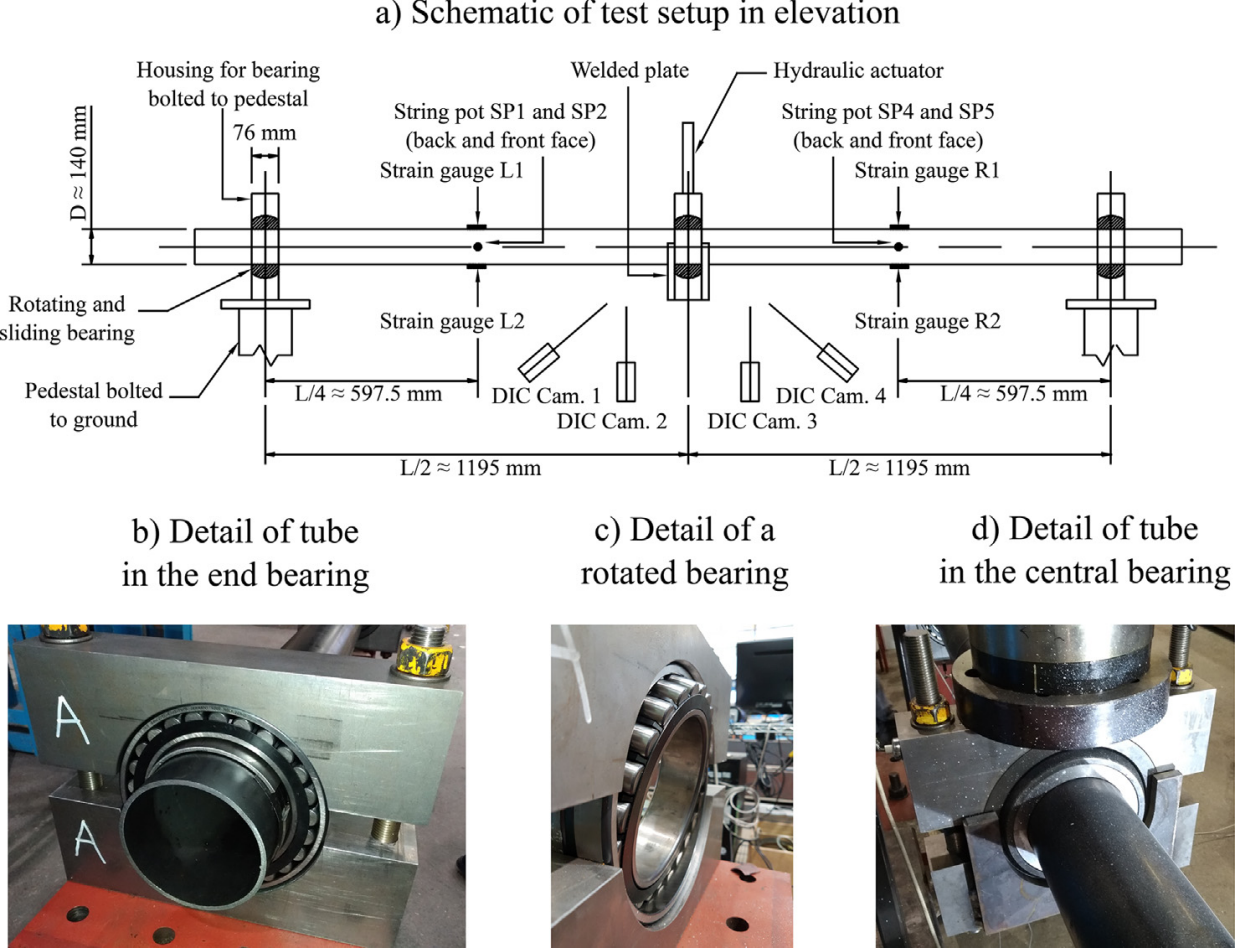
– Schematic of the three-point cyclic bending test arrangement with instrumentation locations and additional details, reprinted from [1] with permission from ASCE [7].

**Fig. 3:**
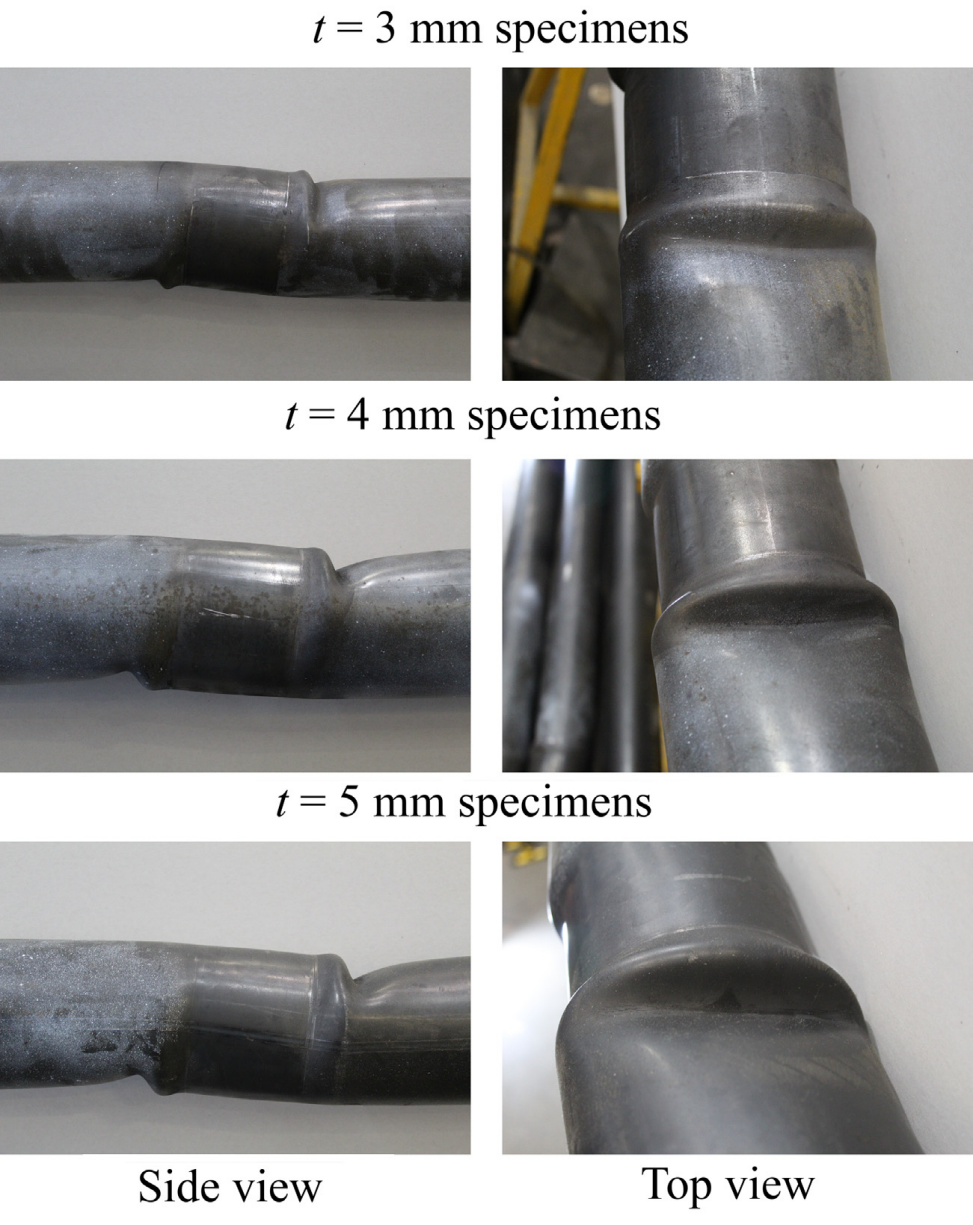
– Representative plastic hinge failure mechanisms for each group of specimens linked by the same nominal wall thickness.

The second data-gathering activity was a series of three-point cyclic bending tests of the tube specimens covering a range of *D*/*t* ratios, seven of which were successfully tested to elastic-plastic buckling failure. Such tests are routinely carried out in structural engineering to establish moment-curvature relationships, and ultimately moment or bending strain capacities, for systems which fail through the formation of a localised plastic hinge. The tests were performed on a custom-constructed rig (see Fig. 2 for a schematic, which also illustrates the placement of measurement instrumentation and details of the support bearings). Fig. 3 illustrates the plastic hinge failure modes for each nominal tube thickness, all relatively similar. The entities measured include:

• Midspan transverse force and displacement at the hydraulic actuator which introduces the load into the tube with the aid of a 99-mm thick and 140-mm wide rigid steel ring;
• Longitudinal surface strains with discrete strain gauges a distance of *L*/4 way from either end support (L1 and L2 to the ‘left’ of midspan, and R1 and R2 to the ‘right’). The time-history records of these strains and the midspan displacements are in ASCII-readable ‘.st’ files;
• Two pairs of DIC systems focusing on a region approximately 300 mm to either side of the load introduction point, with images taken at 10-second intervals and processed using the DaVis v 8.4.0 commercial imaging software. The raw data obtained in this way is far too voluminous to be meaningfully released, and the ‘.dic’ files available in the “Processed test and simulation data” folder represent an already heavily processed version of this data into arrays of (*y,z*) wall position coordinate pairs at different time points during the test (*y* representing the longitudinal coordinate along the tube axis, *z* representing the transverse coordinate), with the origin at the midspan tube centroid.

The third data-gathering activity concerns the finite element simulations of the tests which were conducted as geometrically and materially nonlinear static cyclic analyses with a dynamic implicit solver assuming quasi-static settings with significant additional damping. The model recreated pertinent features of the tests in the following manner:

• The central rigid ring through which the test introduced the loading into the tube was represented as a region of the mesh that was kinematically constrained to a centroidal reference point on which displacements were enforced following the Fulmer et al. [6] cyclic loading protocol. This ensured that this portion of the tube maintained diametral circularity.
• The rigid end bearings were similarly modelled as a region of the tube circumference that was kinematically tied to a centroidal reference point on which beam-like ‘horizontal roller’ boundary conditions were defined.
• A ‘combined’ kinematic material model was defined with material tensile data input interpreted as ‘half-cycle’ data and a number of backstresses set at 5, a value obtained by trial and error.
• The geometry was assumed to be ‘perfect’ and represented by linear four-node reduced-integration S4R shell elements with no midsurface imperfections.

**Fig. 4:**
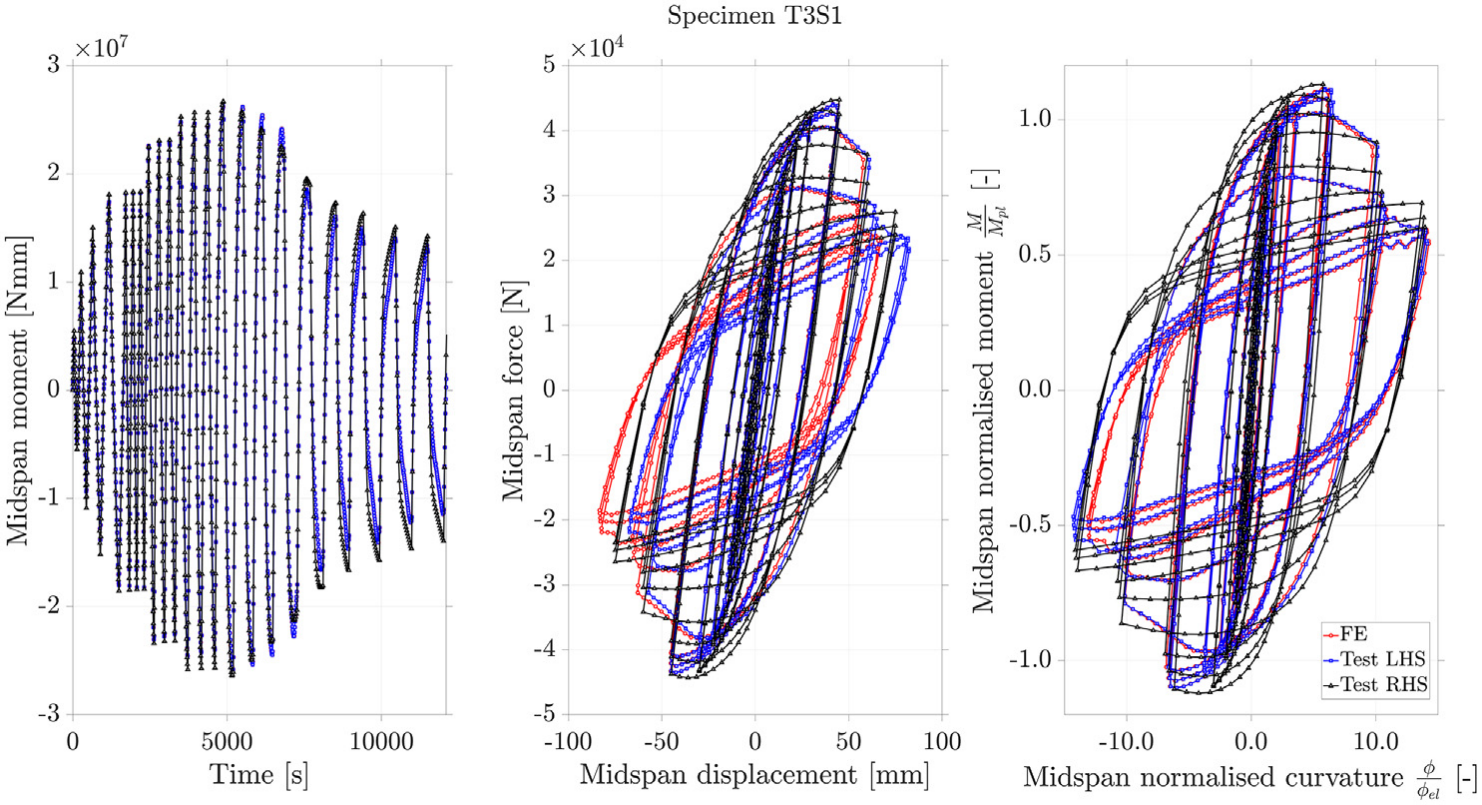
– Moment-time, midspan force-displacement and normalised moment-curvature relationships obtained experimentally (test, using data sampled both to the ‘left’ and ‘right’ of midspan) and by finite element simulation under the assumption of midspan symmetry. The source file for this figure, and for similar figures corresponding to other specimens, may be found in the dataset [2].

The output of the simulations was processed into ‘.st’ and ‘.dic’ files which had an identical data structure to those holding test data. This helped to maintain a consistent dataset where both tests and their simulations were treated in an identical way by the MATLAB processing scripts, aiding in visualisation and interpretation. While both the .st and .dic files generated by the test and simulation data are human readable, it is strongly advised to employ the ”process_st_dic_files.m” MATLAB script to process them simultaneously into informative response-history plots for the midspan moment and hysteresis relationships. The “comparison_figures.m” MATLAB script may then be used to pair the data for a particular test and its simulation, a crucial way to judge the quality of the simulation. An example of a representative relationship generated in this manner is presented in Fig. 4 for the first 3-mm thick specimen (‘T3S1’).

Analysts attempting to reproduce these results should be aware that the model assumptions of symmetry about the midspan loading point are only representative of the response of the cyclically loaded tube *prior* to elastic-plastic buckling, after which each test developed a strongly unsymmetrical response (Fig. 3). It is encouraged that analysts explore different symmetry arrangements (and lack thereof) and trial different shell, continuum-shell and solid continuum elements and kinematic hardening material models to improve the match with the tests.

## Limitations

The main limitation of the dataset of cold-formed S355J2H tube specimens, as far as its representativeness of circular hollow marine piles used in full-scale construction is concerned, is the issue of geometric scale. The tested specimens are in a representative nominal dimensionless *D*/*t* range of 28 to 47, however with an absolute diameter of ∼140 mm and thicknesses between 3 and 5 mm they are significantly smaller than full-scale marine piles. This limitation is as a result of the constraints placed on the project for it to be feasible at the laboratory scale in the available time and budget with a test rig supporting a maximum actuator force (1 MN), stroke (75 mm) and internal roller bearing diameter of 140 mm. Additionally, the *D*/*t* achieved on the basis of the *measured D/t* is 30 to 45, somewhat less than the 28 to 47 range obtained on the basis of nominal geometries, which contributes to the relatively similar sets of behaviours exhibited by all of the specimens including failure modes (Fig. 3). The vexed issue of scale representativeness is, sadly, ubiquitous in experimental structural engineering.

The cyclic finite element simulations made use of a kinematic strain hardening model that was calibrated only on monotonic ‘half-cycle’ tensile material tests instead of (significantly more involved) cyclic material tests which could not be performed in the time available for the project. Despite this, the finite element model as released offers a remarkably good reproduction of the cyclic bending tests. Researchers are welcome to improve on this. Lastly, it was regrettably not possible at the time to extend the (already hours-long) tests to reach a state of full rupture failure in fatigue.

## Ethics Statement

The work undertaken for the purposes of data collection was performed at the Structures Laboratory of Imperial College London. The data collection did not involve human or animal subjects, nor social media platforms, and adheres to the ethical requirements necessary for publication in Data in Brief.

## CRediT authorship contribution statement

**Adam Jan Sadowski:** Conceptualization, Methodology, Formal analysis, Data curation, Writing – original draft, Supervision, Funding acquisition, Project administration. **Wei Jun Wong:** Software, Formal analysis, Data curation, Visualization, Writing – review & editing. **Sai Chung Simon Li:** Software, Formal analysis, Data curation, Visualization, Writing – review & editing. **Christian Málaga-Chuquitaype:** Conceptualization, Methodology, Writing – review & editing, Supervision, Funding acquisition, Project administration.

## Data Availability

Dataset on the tested and simulated response of thick cold-formed circular hollow sections under cyclic loading (Original data) (Figshare).
